# Online learning for orientation estimation during translation in an insect ring attractor network

**DOI:** 10.1038/s41598-022-05798-4

**Published:** 2022-02-25

**Authors:** Brian S. Robinson, Raphael Norman-Tenazas, Martha Cervantes, Danilo Symonette, Erik C. Johnson, Justin Joyce, Patricia K. Rivlin, Grace M. Hwang, Kechen Zhang, William Gray-Roncal

**Affiliations:** 1 The Johns Hopkins University Applied Physics Laboratory, Laurel, MD 20723 USA; 2 Janelia Research Campus, Howard Hughes Medical Institute, Ashburn, VA 20147 USA; 3 Kavli Neuroscience Discovery Institute, Johns Hopkins University, Baltimore, MD 21205 USA; 4 Department of Biomedical Engineering, Johns Hopkins University, Baltimore, MD 21205 USA; 5 Department of Neuroscience, Johns Hopkins University, Baltimore, MD 21205 USA; 6 Department of Computer Science, Johns Hopkins University, Baltimore, MD 21218 USA

**Keywords:** Computational neuroscience, Neural circuits, Synaptic plasticity

## Abstract

Insect neural systems are a promising source of inspiration for new navigation algorithms, especially on low size, weight, and power platforms. There have been unprecedented recent neuroscience breakthroughs with *Drosophila* in behavioral and neural imaging experiments as well as the mapping of detailed connectivity of neural structures. General mechanisms for learning orientation in the central complex (CX) of *Drosophila* have been investigated previously; however, it is unclear how these underlying mechanisms extend to cases where there is translation through an environment (beyond only rotation), which is critical for navigation in robotic systems. Here, we develop a CX neural connectivity-constrained model that performs sensor fusion, as well as unsupervised learning of visual features for path integration; we demonstrate the viability of this circuit for use in robotic systems in simulated and physical environments. Furthermore, we propose a theoretical understanding of how distributed online unsupervised network weight modification can be leveraged for learning in a trajectory through an environment by minimizing orientation estimation error. Overall, our results may enable a new class of CX-derived low power robotic navigation algorithms and lead to testable predictions to inform future neuroscience experiments.

## Introduction

A fundamental problem facing both autonomous robots and biological organisms is the task of navigating complex and time-varying environments. In the robotics field, this is a long- standing problem that has inspired decades of research into increasingly powerful approaches^1^. Critical components of navigation approaches include state estimation of the robot using sensor data and sensor fusion, including classic approaches such as Kalman filtering, along with its nonlinear extensions^2,3^, as well as loop closure, where storage of processed sensory data is used to recognize the return to specific locations in an environment^4,5^.

Real time state estimation for compact platforms, such as small drones, remains dominated by visual odometry due to the power and footprint requirements for more computationally intense algorithms^6^. Many visual odometry approaches for state estimation exist^7^, including high performing Visual-Inertial Odometry (VIO) systems for state estimation on rapidly moving platforms^6^. In general, these approaches require simplifying linearization assumptions^8^, which can be addressed with more computationally intensive approaches such as particle filtering^9^. Nonlinear approaches which adapt to changes in the environment remain an ongoing research challenge. Additionally, visual odometry approaches do not perform loop closure and are prone to error accumulation when used in isolation. In particular, observed visual features that have been previously encountered are not used to update pose estimation.

Algorithms for visual approaches to Simultaneous Localization and Mapping (SLAM), which perform loop closure, are extensively used on higher power platforms^10–13^. Real time, efficient implementation of visual SLAM systems on embedded mobile platforms remains a major engineering challenge^14,15^, often requiring custom hardware designs for bundle adjustment and loop closure^16^. There are also emerging visual navigation and visual SLAM approaches based on deep learning, including self-supervised approaches^17^. This has resulted in powerful approaches for monocular visual odometry^18^, and also novel strategies such as vector navigation on representations with grid-cell like responses^19^ and predictive navigation in complex environments^20^. Promising approaches in deep learning for unsupervised training of visual odometry approaches include the intermediate calculation of depth from images, which can be refined in tandem with pose estimation by offline network optimization on a collected dataset^21^. In general, however, training of the neural networks utilized in visual navigation are not performed during robotic system deployment due to computational requirements for training and the number of required training samples.

Neuromorphic processing is a promising path forward for embedded neural network navigation approaches considering the ability to process data from low-latency event-driven sensors, perform on-chip training, and execute power-efficient computation. Event-driven cameras and processing have been shown to produce low-latency, high-performance VIO systems for quadcopters^22^. The development of general purpose neuromorphic hardware, such as the spinnaker^23^, IBM’s TrueNorth chip^24^, and the Intel Loihi^25^ enable the decoupling of neuromorphic algorithm design from custom hardware implementation. The Loihi is particularly well-suited for robotics applications, as it allows on-chip learning and has demonstrated efficiency in the classification of dynamic data in applications such as keyword spotting^26^ and olfactory signals^27^. Given the emergence of platforms, such as Loihi, with well-documented power efficiency and where networks can be deployed with flexible online learning rules, a challenge for neuromorphic robotic approaches is the identification and development of underlying algorithms. Navigation strategies utilizing event-driven data and neuromorphic processing are particularly compelling for their potential to provide low-latency, adaptive navigation solutions with efficient implementations which motivates further algorithm development.

Biological systems are a natural source of inspiration for navigation algorithm development for neuromorphic processing, considering their navigation capabilities and low power utilization. Mammalian systems are among the most studied in neuroscience in relation to navigation with research breakthroughs over the past decades identifying several specialized cell types including head direction cells^28^, grid cells^29^, and place cells^30^. There have been several approaches utilizing these representations and additional specialized mammalian cell types^31,32^ to propose algorithms for neuromorphic robotic navigation^33–38^. While mammals have been implicated in map-based navigation strategies^39^, in insects, with drastically reduced nervous system sizes, place-cell like representations have not been identified and it is unknown if similar underlying cognitive map-like representations are calculated^40^.

Head-direction cells are found across different mammalian species^31^ as well as non-mammalian species such as the fly, *Drosophila melanogaster*^41^. The head-direction cells are conceptually the simplest among the spatial neurons because the internal representation is only one-dimensional with ring topology. Currently the leading theoretical models for the head-direction cells are ring attractor networks^42–44^. Although many models have been proposed^45–48^, they all share the same basic principle of using a stable equilibrium state of a ring network, which we call an activity bump, to represent the current internal sense of direction. The input from angular velocity of head movement serves to drive the activity bump around the ring of cells, and the input from sensed landmarks serves to anchor the peak position of the bump to the learned position. Although the mammalian head-direction systems have been intensely studied in the ensuing decades since their initial discovery, connections from the visual or vestibular systems to the conceptual ring topology have to go through multiple bilateral structures^49,50^; consequently the detailed circuit diagram (i.e., connectome) has never been established. Thus, these theorized networks require a large set of design decisions often with minimal constraints from experimental observations or known anatomical connectivity. In particular, existing robotic studies inspired by the mammalian head direction systems all require solutions for head direction error accumulation with angular velocity integration without connectivity constraints at an observed cell-type level^35,51–56^. One series of approaches to prevent error accumulation is to reset estimates at a signal derived from a particular orientation^52^, reset at regular time intervals from another reliable heading direction measurement^36^, or to utilize a set of visual input features active at particular discrete orientations^57^. In the absence of such well-defined external orientation cues, other approaches implemented for robotic systems include utilizing learning between higher-level map-like representations at mammalian-inspired place cells to learn the correspondence between sensory input and a heading direction^38^. Additionally, the parallax of close landmarks, which have sensed visual features that vary with position (as opposed to landmarks at an infinite distance on the horizon), has been less well studied in mammalian head direction computational models^58^ and represents a necessary challenge in creating an orientation estimation system in cluttered environments during translation.

Compared to mammalian systems, insect systems are a compelling source for navigation algorithm development because of their low size, weight and power (SWaP), the relative compactness of the networks, and an enhanced mechanistic understanding, where the detailed connectivity patterns are known. Recent experimental breakthroughs in *Drosophila* have identified a candidate set of “compass” neurons involved in a ring attractor network for representing heading direction in a neural region called the central complex (CX). Elucidating the structure and function of *Drosophila* is an active area of study and large-scale wiring diagrams are being reconstructed, providing a rich substrate for analysis, model refinement and validation^59–61^. Compared to the theoretical plausibility of a ring attractor for heading direction in mammalian systems, experiments have observed and quantified an activity bump corresponding to the insect’s heading direction^41,62^. Furthermore, neurons responsible for providing input into the ring attractor for angular velocity^63,64^ and visual features^65,66^ have been characterized experimentally. Correspondingly, the detailed anatomical connectivity patterns between neurons involved in the network have been observed^67^ and investigated for their computational role^68–70^ at the level of individual neuron type to support ring attractor dynamics. A unique opportunity exists, therefore, to derive mechanistic inspiration for a navigation system utilizing detailed connectivity patterns observed in *Drosophila* for representing heading direction in a ring attractor network.

In order to utilize visual landmark cues from an environment for orientation estimation, a mapping must be learned between the location of visual features on a sensor to an orientation referenced to a world frame coordinate system. Theoretical models have been proposed for how Hebbian unsupervised learning can lead to the strengthening of connections between neurons receptive to visual features and heading direction ring attractor neurons that are co-active in order to learn the mapping between landmark cues and an orientation estimate^42,71^ . Hebbian learning is a general type of synaptic mechanism for associative learning well studied in neuroscience and recent experimental evidence in *Drosophila* support a role for Hebbian learning for the mapping between neurons representing visual features and the compass neurons^66,72^, however the performance of such Hebbian learning is unknown with changes of position, where the relative landmark angles change over time.

While there has been compelling insight into the *Drosophila* heading direction system at a cell-type level, an outstanding question is how amenable this approach may be for robotic applications that include a trajectory through an environment. In particular, the performance of the heading direction system has not been quantified when using cell-type specific connectivity patterns. Additionally, due to constraints of experimental approaches, both empirical observations and existing computational models have been limited to single location trajectories where there is rotation without translation. In particular, the coordinated learning of a visual feature mapping while traversing a trajectory through an environment is a necessary and challenging function for such a network that is not currently understood.

The key contributions of this work are to develop and evaluate a model for how a *Drosophila* connectivity-constrained network can perform both sensor fusion and online learning for estimating orientation in a trajectory through an environment to better understand the mechanisms employed in biology and to enable neuromorphic visually guided robotic navigation. We evaluate the model through trajectories with noisy inputs in a simulated environment, investigate performance with measurements from a robotic platform, and quantify the potential power efficiency gains of neuromorphic implementation with such a compact network model. Furthermore, we propose a theoretical understanding of how distributed online unsupervised learning can be leveraged for learning in a trajectory through an environment in coordination with a ring attractor network that addresses current challenges in deployment time training of neural networks as part of a navigation algorithm.

## Results

A connectivity-constrained network model with online learning is developed and evaluated with a series of targeted experiments to investigate (1) the coordination of insect cell-type connectivity with online learning, (2) the learning challenge where the relative orientation of visual cues changes over time with changing position throughout an environment, and (3) network performance when utilized on a robotic platform. Finally, we analyze the network’s online learning from the perspective of objective function minimization and propose an updated online rule to enhance heading direction estimation accuracy which can be investigated further in behavioral experiments.

### Connectivity-constrained network for sensor fusion and online learning

**Figure 1:**
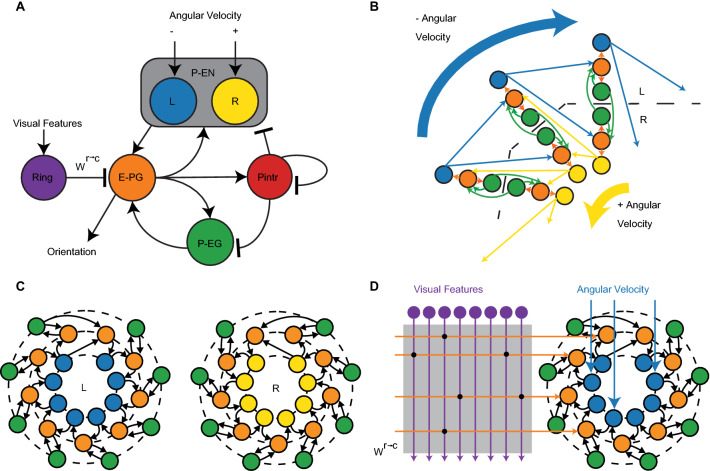
*Drosophila* CX ring attractor model for sensor fusion and environmental learning. (**A**) Five neuron types found in the *Drosophila* central complex (CX) included in the ring attractor model. Input is provided to the model as visual features to the ring neurons and angular velocity to the P-EN neurons. P-EN neurons are preferentially activated by positive and negative angular velocities according to if they are located in the left or right hemisphere of the PB. Orientation is encoded in E-PG compass neurons. All neuron types are excitatory except for Pintr and ring neurons which provide inhibition. (**B**) The primary repeated pattern in the connectivity constrained ring attractor network organized by E-PG neuron projecting to glomeruli in the left and right hemisphere of the PB. Local recurrent excitation enables the sustained activation of an activity bump, with positive or negative angular velocities shifting the center of the activity bump across the ring attractor network. (**C**) The connectivity pattern utilized for all excitatory modeled neurons separated by PB hemisphere. Note that there is one additional modeled E-PG neuron per hemisphere than other neuron types. (**D**) A weight matrix, ${{\varvec{W}}}^{{\varvec{r}}\to {\varvec{c}}}$, is learned, which specifies the strength of connections between ring neurons and compass neurons.

Our model transforms angular velocity and visual features into a fused representation of orientation utilizing a total of 141 neurons distributed across five populations of neurons as specified in Fig. 1A, which are constrained by connectivity patterns of neuron types observed in *Drosophila* across glomeruli, and with plastic synaptic connections that enable the learning of visual landmarks. The five types of modeled neurons all synapse in either the protocerebral bridge (PB) or ellipsoid body (EB) regions of the CX and include: (1) Ring neurons which are receptive to visual inputs, (2) PB-EB-Noduli (P-EN) neurons which receive angular velocity inputs, (3) EB-PB-Gall neurons (E-PG) neurons, (4) PB-EB-Gall (P-EG) neurons, and (5) intrinsic neurons of the PB (PIntr), also referred to as Δ7 neurons. Orientation is encoded in a population of E-PG neurons or “compass” neurons, which have been observed in experimental studies to have maximal activation in a position along the anatomical circumference of the ellipsoid body which rotates corresponding to the orientation of the fly. The modeled population of 18 E-PG neurons is divided into left and right hemispheric groupings depending on whether the neuron projects to the left or right hemisphere of the PB. We utilize an orientation representation scheme where each of the 9 E-PG neurons per hemisphere is maximally active at a distributed preferred angle as demonstrated in Fig. 1C. The architecture of our model is constrained by the nine anatomically observed glomeruli observed per hemisphere in the PB representing a discretization in the heading direction system uniquely observed in insect systems^63^.

A stable bump of activity in E-PG neurons can persist given the recurrent excitation to keep the bump active along with inhibition provided by PIntr neurons to prevent runaway excitation. Local recurrent excitation for maintaining bump activation in E-PG neurons is mediated by both P-EN and P-EG neurons. Notably, the PIntr neurons do not directly inhibit E-PG neurons, but rather inhibit the P-EN and P-EG as well as themselves in a more complex pattern of inhibition than would be strictly necessary to prevent runaway excitation among the E-PG neuron population. Positive angular velocity is encoded in the P-EN neurons in the right hemisphere, which when active, shifts the activity bump in the counter-clockwise direction. Conversely, negative angular velocity is encoded by P-EN neurons in the left hemisphere which shifts the activity bump in the clockwise direction (Fig. 1B).

Sensed visual features represented in ring neuron activation patterns can update the activity bump in E-PG neurons according to their connectivity weights (Fig. 1D). The intuition behind the online learning of visual features is that the activity bump is initially driven by angular velocity signals which serves as a noisy teaching signal to update a matrix of weights, ${W}^{r\to c}$, between ring neurons and “compass” E-PG neurons. Each individual element, ${w}_{nm}$, is the weight between the mth ring neuron and the nth E-PG neuron. The mechanism utilized for learning is a Hebbian learning rule, where the co-activation of neurons increases the effective connection weights between ring and E-PG neurons. In particular, a presynaptically-gated Hebbian learning rule is used to modify weights^72^,

1 $$\Delta w_{nm} = \eta r_{m} \left( {{\text{a}}c_{n} + {\text{b}} - w_{nm} } \right)$$

where $r_{m}$ is the activation of the mth ring neuron, and $c_{n}$ is the activation trace of the n^th^ E-PG neuron. The rule is parameterized with a learning rate, $\eta$, as well as additional constants $\mathrm{a}$ and $\mathrm{b}$. All utilized ${w}_{nm}$ weights are assumed to be negative, as in previous computational studies^68^ given the inhibitory characteristics of ring neurons^66,73^, so the maximum effective weight is zero, which can activate E-PG neurons through disinhibition. When the network model is deployed for a simulated or physical environment, all other weights besides ${W}^{r\to c}$ are held fixed. The model’s orientation estimate is calculated as the center of the activity bump utilizing the preferred direction of each E-PG neuron to create a linear decoder applied to filtered spiking events. Additional details for model implementation can be found in the methods section.

### Model performance with rotation

**Figure 2:**
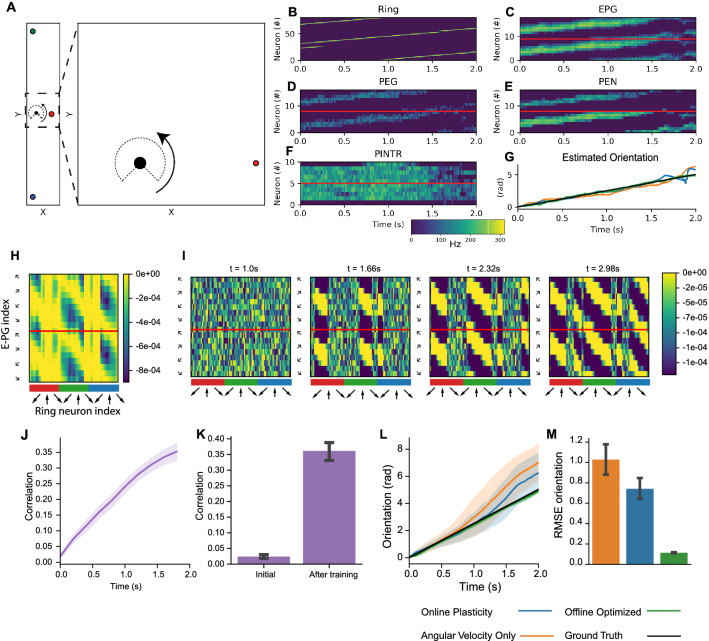
Sensor fusion and online learning with rotation in simulation. (**A**) Visual and angular velocity inputs to network correspond to a rotation in a simulated environment with landmarks and a 270° field of view. (**B**–**F**) Neuron activation over time in each population supports sensor fusion of inputs into an activity bump that shifts in the E-PG neuron population during rotation. **(G)** Orientation estimation decoded from E-PG neuron activity (best seed example) with evaluated model configurations tracks the ground truth orientation. (**H**) Offline optimized ${{\varvec{W}}}^{{\varvec{r}}\to {\varvec{c}}}$ specifies an optimal mapping from visual landmarks encoded in ring neurons to E-PG “compass” neuron activation. Ring neuron indices are arranged sequentially according to receptive field center for each colored landmark. (**I**) In the model configuration with online plasticity, weights evolve over time to a similar structure as offline optimized weights. (**J**–**K**) Correlation between online learned weights and the offline optimized solution increases over time. (**L**) Estimated orientation over time drifts less with online plasticity then with angular velocity alone. (**M**) Average orientation RMSE. All group level analyses in (**J**–**M**) are performed for 50 simulated seeds. (**J, K, M**) have 95% bootstrapped confidence bounds, (**L**) has standard deviation confidence bounds.

An initial evaluation of the network is performed in the case of a simple rotation in an environment without translation (Fig. 2). Ring neurons encode visual input, with a separate set of ring neurons responsive to each landmark and each individual ring neuron with a receptive field tiled across a 270° field of view. As the simulated agent and field of view rotates, the index of the most active ring neuron in each sub-population shifts (Fig. 2B). A corresponding shift in a bump of activity is observed in E-PG, P-EN, and P-EG neurons (Fig. 2C–E), which appears as two bumps given the separate indexing of left and right hemisphere neurons. Angular velocity input is provided to the model by injecting current in the P-EN neurons, where a counter-clockwise rotation in the simulation corresponds to an observed increase in activation of right P-EN neurons (Fig. 2E). Throughout the trial, there is consistent activation of Pintr neurons (Fig. 2F), which provide inhibition to the network and prevents the model from going into a state of runaway excitation.

Three model configurations are compared in order to evaluate sensor fusion and online learning (1) initialization of ${W}^{r\to c}$ weights to an optimal set of offline calculated weights, (2) online learning of modeled weights according to Eq. (1) after random initialization, and (3) setting all ${W}^{r\to c}$ weights to zero such that angular velocity is the only input into the model. Due to the stochasticity of the neuron model, a total of 50 simulation seeds are used to evaluate performance. An example of the decoded orientation from all three model configurations is shown in Fig. 2G, where all of the model configurations are able to track the orientation over time (with the optimal offline weights calculated weights having the best performance). The optimized weights (Fig. 2H) are calculated with regularization, which effectively select a subset of model ring neurons to utilize for angle estimation. The weights that are learned over the course of the trial (Fig. 2I), have a similar banded structure to the optimized weights and increase in correlation over the course of the trial to an average value of 0.36 (Fig. 2J). Factors limiting higher correlation include (1) the effective “teaching” signal in the online training is from integrating angular velocity cues which are inherently noisy as well as (2) the inherent differences between the training approaches. Estimated orientation error accumulates over the course of the trial in the angular velocity only case (Fig. 2L), which is reduced when there is online learning. Overall, the average orientation root mean squared error (RMSE) is significantly reduced by 28% (1.03 to 0.74 radians, P = 0.0023, Mann–Whitney *U*-test) with the online learning verses angular velocity alone, compared to an 89% reduction with the set of optimized weights. Sources of error in the weight optimization process include: (1) ${W}^{r\to c}$ is optimized with regards to the feedforward visual input alone and target E-PG activity which does not explicitly compensate for the recurrent activation from P-EG and P-EN neurons, (2) there is an inherent time lag in spiking neuron activation given the 20 ms modeled time constant with the leaky integrate and fire neurons, (3) the orientation is decoded from an estimate of the center of the bump from spiking activity of 18 neurons. Nevertheless, a RMSE of 0.11 radians (6.3°) in the optimized weights demonstrates that this network with a limited number of spiking neurons and recurrent excitation can effectively perform sensor fusion for accurate orientation estimation.

### Model performance with translation

**Figure 3:**
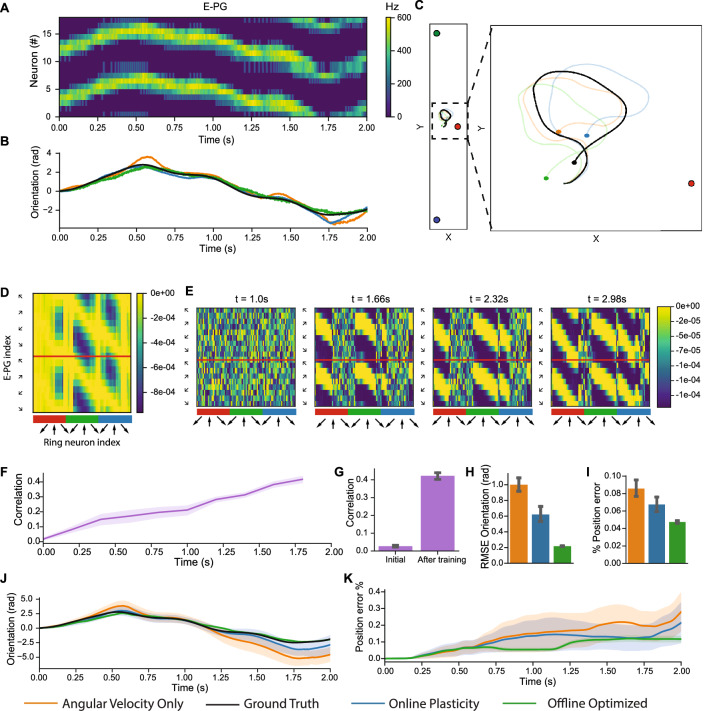
Sensor fusion and online learning with translation in simulation. (**A**) Shifting of the activity bump in E-PG neurons in the online learning model corresponds to changes in orientation. (**B**) The decoded orientation estimation (best seed) with model configurations tracks the ground truth trajectory. (**C**) Two distant landmarks and one close landmark provide visual inputs to the network. Position estimates with path integration approximate ground truth (final positions denoted with a circle). (**D**) Offline optimized ${{\varvec{W}}}^{{\varvec{r}}\to {\varvec{c}}}$ has features preferentially tuned to distant landmarks. (**E**) Evolution of weights during online plasticity share structure with offline optimized weights. (**F–G**) Correlation between online learned weights and the offline optimized solution increases over time. (**H**) Average orientation RMSE with online plasticity is less than angular velocity alone model configuration. (**I**) Improvement in position error (average position error as a percentage of path length) additionally observed with online plasticity versus angular velocity alone. In orientation estimates (**J**) and position error as a fraction of path length (**K**), error accumulates less with online plasticity than angular velocity alone. Group level analyses in (**F**–**K**) are performed for 50 simulated seeds. (**F**–**I**) have 95% bootstrapped confidence intervals, (**J**–**K**) have standard deviation confidence intervals.

The network is next evaluated in a more challenging trajectory that involves translation through the simulated environment, where there is no longer an invariant transformation between egocentric visual landmark features referenced to a sensor frame and an orientation referenced to an allocentric world frame coordinate system (Fig. 3). The bump of E-PG activation follows the time-varying orientation in the trajectory and is able to be decoded into an accurate orientation estimation over time (Fig. 3A–B). In the trajectory through the environment, there are two distant landmarks, which individually have a relatively invariant transformation between egocentric and allocentric representations, along with a proximal landmark which has a time-varying transformation between egocentric and allocentric representations (Fig. 3C).

The set of offline optimized weights for the network (Fig. 3D) preferentially selects visual features corresponding to the distant landmarks. The evaluated network configuration with online plasticity learns weights with increasing correlation over time to the optimal weights (Fig. 3E–G). Similar general trends for orientation estimation accuracy are observed between network configurations as to the rotation only trajectories (Fig. 3H, J), where the orientation RMSE for online learning is between the angular velocity only case and the optimal set of weights. The RMSE of the angular velocity only network configuration in this trajectory with translation is similar to the simple rotation case (1.00 vs. 1.03 radians), but is compensated further by online learning (38% vs. 28% reduction in error).

Position estimation with path integration is more challenging than orientation estimation because of the accumulation of error driven by orientation estimation error. Similar trends are observed with average position estimation error (as a percentage of path length), where online learning has a position estimation error between the angular velocity alone and the optimized weights network configuration (6.7% vs. 8.6% and 4.8%, Fig. 3I). The decrease in average position error with online learning vs. angular velocity alone is 22% (*P* = 0.0021, Mann Whitney U-test). Sources of error for position estimation for path integration include the aforementioned error accumulation from orientation estimation that could be further reduced with neuro-inspired^74^ or traditional approaches^75^.

Overall, network simulation results with translation demonstrate that an accurate allocentric estimation of orientation referenced to a world frame can be estimated with egocentric visual features despite the challenges of transforming information over time from proximal landmarks. The model configuration with online learning is correlated with the optimized set of weights and improves orientation and position estimation by an average of 38% and 22% respectively versus a configuration with angular integration alone. Simulation results suggest that improvements to the online learning rule (Eq. 1) may be beneficial to effectively select visual features from distal landmarks to further increase accuracy.

### Model performance on a robotic platform

**Figure 4:**
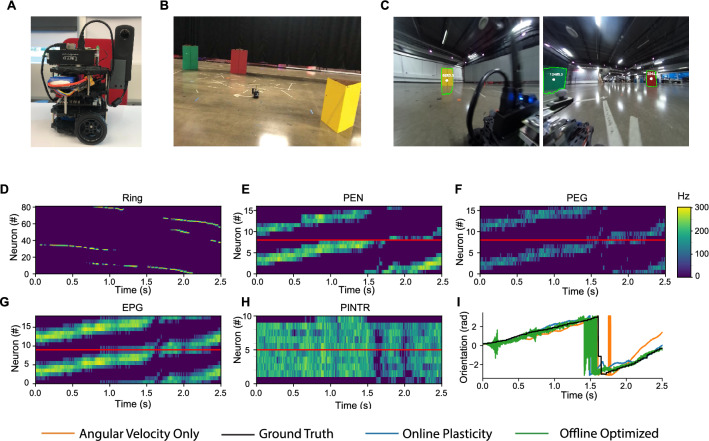
Model utilized with a robotic platform. (**A**) Modified Robotis Turtlebot3 “Burger,” equipped with a Nvidia Jetson TX2, and a Ricoh Theta S 360° camera. (**B**) Physical arena with colored landmark cues. (**C**) Example camera measurements with blob detection utilized to drive activation of visual neurons. (**D–H**) Neuron activation over time in each population driven by processed sensory data from the robotic platform, where visual landmark features encoded in ring neurons (**D**) drive shift in activity bump in E-PG neurons (**G**) in model configuration with online plasticity. (**I**) The estimated orientation from each model configuration (best seed) tracks the ground truth orientation with errors in the offline optimized weights at periods of prolonged visual dropout.

The model for orientation estimation and position estimation is extended from simulated environments to measurements from a wheeled robotic platform (Fig. 4) in an arena with colored landmarks (Fig. 4B). Visual inputs are measured from a camera with two separated sensors offset at 180°, where the relative angle offset of landmarks is calculated from blob detection on color-masked images (Fig. 4C). The relative angle offset detected for the center of each of the green, yellow, and red colored landmarks are used to drive the activation of ring neurons (Fig. 4D) according to their receptive fields, which are mapped equivalently to the three populations of ring neurons mapped to simulated landmarks across a 270° field of view. Given the less than 360° field of view on the camera sensors, there are ring neurons selective to outside the camera’s field of view which will never activate. While several different neural visual encoding schemes are possible which would likely improve performance, mirroring the simplified visual encoding from simulations enables straightforward comparison between simulated and physically measured model performance. An added source of noise in the visual feature measurements is the intermittent dropping of recorded image frames due to maximum disk-writing speeds, which is apparent in the lack of ring neuron activity at intermittent periods, which further tests the network’s performance in relation to sensor fusion. The angular velocity measurements to drive the activation of P-EN neurons (Fig. 4E) are derived from an on-board IMU sensor.

**Figure 5:**
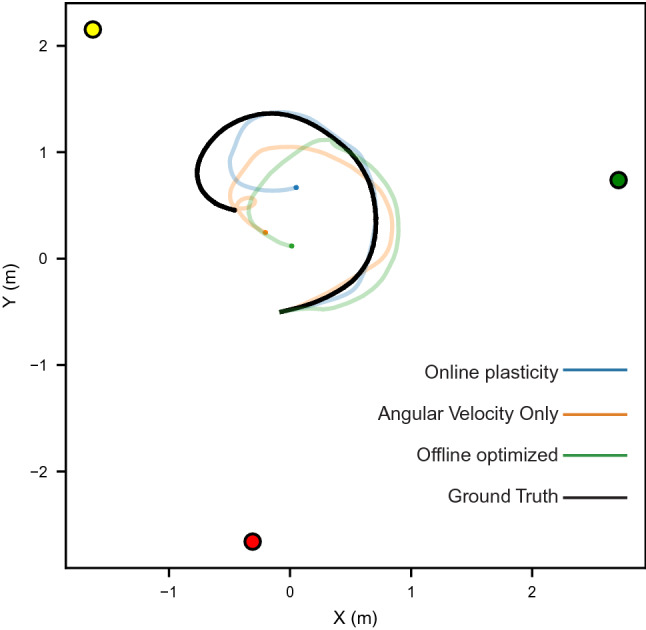
Trajectory estimation. In the arena, position estimates from each model configuration share features of the overall trajectory (best seed) with the ground truth trajectory. Landmark positions are denoted with colored circles. Final positions in the trajectory estimates are denoted with a small circle.

**Figure 6:**
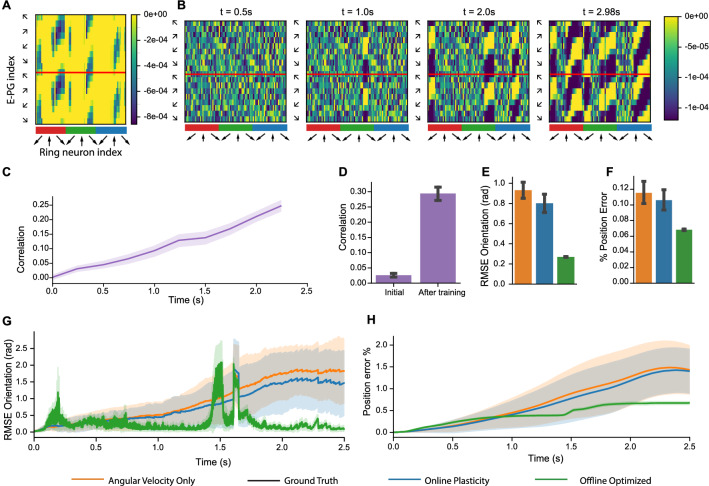
Model evaluation on a robotic platform. (**A**) Offline optimized ${{\varvec{W}}}^{{\varvec{r}}\to {\varvec{c}}}$ has a banded structure which represents an optimal set of weight to map visual features in ring neurons to orientation representation in E-PG neurons. (**B**) Evolution of weights during online plasticity increases in similarity to offline optimized weights over time. (**C–D**) The correlation between online learned weights and the offline optimized solution increases over time. (**E**) The average orientation RMSE (in radians) is less with online plasticity than angular velocity alone. (**F**) Average position error as a percentage of path length has similar trends as orientation estimation with less differentiation between online learning and angular velocity alone. (**G**) In estimated orientation RMSE over time, error increases in periods with visual feature dropout with offline optimized weights. (**H**) Position error as a percentage of path length over time. All group level analyses in (**C–H**) are performed for 50 simulated seeds. (**C–F**) have 95% bootstrapped confidence intervals, (**G–H**) have standard deviation confidence intervals.

The center of the bump of activity in the P-EG and E-PG neurons shifts over time (Fig. 4F–G), which leads to a decoded orientation that follows the ground truth orientation (Fig. 4I) and a position estimate utilizing ground truth linear velocity for path integration (Fig. 5). A comparison of the model performance is performed with the plasticity model, the angular velocity only model, and the optimized weights (Fig. 6). An example of the comparison between the optimized weights and the online learning of weights is shown in Fig. 6A–B, which again share a banded structure. The correlation of the optimized and online learned weights monotonically increases over time to a maximum average value of 0.29 (Fig. 6C–D). The orientation error accumulates over time fastest in the angular velocity only case with a slight decrease in error accumulation with online learning (Fig. 6G). Overall, the orientation RMSE is 14% less with online learning vs. angular velocity alone (average 0.80 vs. 0.93 radians, *P* = 0.014, Mann–Whitney *U*-test), however the optimized weights have the lowest error. The position estimate similarly accumulates over time in all comparisons, with a notable increase in position error at 1.5 s in the weight optimized case due to a prolonged period of dropout of visual features (Fig. 6H). A minor decrease in the average position error with the online learning versus the angular velocity case alone is observed.

To analyze the potential power efficiency impact for neuromorphic implementation, we can estimate the amount of power required for the algorithmic computation of our model based on reported estimates from SPICE simulations of Intel’s Loihi neuromorphic chip^25^. Utilizing figures for energy per synaptic spike operation, synaptic update, neuron update, and within-tile spike energy, we can approximate the power utilization necessary for the algorithmic computation of our model on neuromorphic hardware as 18.58 μW (11.42 μW for neuron update, 4.38 μW communication, and 2.77 μW for plasticity).

### Online learning analysis

In all experiments, weights derived from online learning are correlated with an optimal solution and improve orientation accuracy after a single trajectory. A challenge for robotic translation is how to enhance estimation accuracy to further minimize error accumulation.

One approach is to identify the objective function that the utilized learning rule is minimizing. Upon further inspection (see “Methods” Eqs. 5–6), it follows that Eq. 1 is minimizing the overall objective function,

2 $$\phi = - a\mathop \sum \limits_{n} c_{n} I_{r \to n} + \frac{1}{2}\mathop \sum \limits_{n} \mathop \sum \limits_{m} r_{m} (b - w_{nm} )^{2}$$

where $I_{r \to n} = \mathop \sum \limits_{m} r_{m} w_{n,m}$. The first term, $- a\mathop \sum \limits_{n} c_{n} I_{r \to n}$, minimizes the objective function when the E-PG neuron activity and the total input current from all of the ring neurons are aligned. The second term, $\frac{1}{2}\mathop \sum \limits_{n} \mathop \sum \limits_{m} r_{m} (b - w_{nm} )^{2}$, effectively acts as a regularization term on the weight values, which is minimized the closer each weight value is to $b$. While the learning rule (Eq. 1) effectively maximizes the overlap from the input current from ring neurons and the E-PG neuron activation, it is not directly optimizing an objective function to minimize the squared error of the orientation estimate. An example of this is how the online learning model (Eq. 1) did not preferentially select distal over proximal landmark features. The success of utilizing a scaled version of the offline optimized weights solution in preferentially selecting distal landmark visual features in simulation (and increasing accuracy overall in simulations and on the robotic platform), lends support to using a set of directly optimized weights that minimize squared orientation error in future approaches. In order to directly minimize the objective function of the scaled squared orientation estimation difference, we can define the objective function for each E-PG neuron,

3 $${{\varphi }} = { }\mathop \sum \limits_{n} \left( {c_{n} - {\upbeta }I_{r \to n} } \right)^{2} = { }\mathop \sum \limits_{n} { }c_{n}^{2} { } - 2c_{n} {\upbeta }I_{r \to n} + { }\left( {{\upbeta }I_{r \to n} } \right)^{2}$$

with a scaling factor, ${\upbeta }$. Upon further inspection, it follows that in order to minimize the objective function,${{ \varphi }}$, (see “Methods” Eqs. 7–8), a weight update rule is

4 $$\Delta w_{nm} = \eta r_{m} \left( {c_{n} - \beta I_{r \to n} } \right)$$

This rule has a commonality to the previously utilized rule (Eq. 1), where weight modifications are presynaptically gated; however, there is an additional term, ${I}_{r\to n}$, that is utilized. We propose the learning rule above (Eq. 4) for utilization in future online robotic applications to enable an improvement in accuracy in learning an environmental mapping. Each of the terms utilized in Eq. (4) is biologically plausible, which could motivate future experimental evaluation. Furthermore, each term is a local variable specific to pairs of synaptically coupled neurons, which would be amenable for implementation in distributed learning on neuromorphic hardware.

## Discussion

Our motivation is to investigate the potential to leverage details from *Drosophila* neurobiology and neuroanatomy for sensor fusion and online learning for orientation estimation as a basis for future low SWaP neuromorphic robotic navigation approaches. Central to our analysis is understanding how online learning in the underlying insect neural circuit can incorporate visual features during changing positions in complex environments, a necessary functionality for both robotic and biological systems. We develop a compact model for *Drosophila* sensor fusion and online learning in a cell-type connectivity-constrained model for orientation estimation and environmental learning that integrates angular velocity measurements and visual features. Through a series of experiments in simulated environments, we demonstrate that improvement in orientation and position accuracy estimation is possible with online learning of visual features (versus angular velocity alone) over a single trajectory and that online learned weights are correlated with a set of offline calculated optimal weights. The network model is adapted for use with sensors from a robotic platform and is demonstrated to have increased accuracy with online learning over a single trajectory. Finally, a theoretical understanding and weight update rule for distributed online learning with local variables is proposed that can be utilized to minimize estimation error.

There are many neuroscience-inspired robotic approaches for navigation that are largely inspired by the cell types observed in mammalian systems, e.g.^33^, with a subset of these utilizing a ring attractor to represent heading direction, e.g.^53^. In comparison to robotic approaches that utilize well-defined external cues^36,52,57^, or projections to mammalian-inspired place cells^38^ to reduce accumulation of error with angular velocity integration in a heading direction network, we demonstrate learning a mapping between neurons encoding visual features over receptive fields to neurons encoding heading direction which has been experimentally observed. We demonstrate that online learning can decrease heading direction estimation error by 14–38% in a single trajectory through the environment by emulating learning that has been observed in *Drosophila* at ring neuron to E-PG neuron synapses^66,72^. A salient difference between insect and mammalian heading direction systems is the far fewer number of neurons that insects have in their heading direction system. Additionally, insect models benefit from the greater level of cell-type connectivity information available, as compared to mammalian systems. Models of ring attractor networks developed for mammalian heading direction estimation typically employ (1) a mechanism for self-sustained activation, (2) direct, non-recurrent inhibition, and (3) representations of heading direction based on large numbers of neurons that approach continuous approximations^42–48^. In insect systems, however, network architectures identified in the heading direction system are more compact but more complex in network details, which include (1) additional paths of recurrent excitation facilitated by an additional class of neurons (P-EG), (2) a distributed population of inhibitory neurons, which do not directly inhibit the compass neurons, and (3) a discretization of the compass neurons into nine glomeruli per hemisphere^40^. Computational studies have been performed outlining how this compact set of *Drosophila* cell types can have properties as a ring attractor^68,70^, but we demonstrate here how the network can be operationalized for sensor fusion and orientation estimation for robotic navigation. Considering that features of this *Drosophila* network are preserved across insects^70,76^, which systemically differ from conventional rodent-based ring attractor networks, the navigation implementation proof of principle established here can inform future studies into enhanced mechanistic understanding and performance of this system.

We investigate how a heading direction system can learn sensory cues to maintain accuracy with a changing position across an environment from the perspective of objective function minimization. Previous computational modeling, focused on a fixed location^42,71,72^, investigated Hebbian plasticity rule formulations which will lead to increasing the effective connectivity weights between co-active visual input feature neurons and compass neurons, but without minimizing an objective function for orientation estimation error. At a single location, our results show that a Hebbian rule is sufficient to learn mappings between landmark features, however when the spatial location is allowed to vary, a Hebbian rule formulation struggles to ignore uninformative landmark features. Indeed, at multiple spatial locations or when there are uninformative landmark features, estimating heading direction from a set of visual features can be challenging^58^, without a set of network weights that can perfectly perform this mapping. Nevertheless, a set of network weights can be identified which minimize an objective function for heading direction estimation from visual features. We find that landmarks at a distance help anchor the heading system better than nearby landmarks through analysis of optimized visual feature weights, which is intuitive because a distant landmark provides a more consistent signal at different positions in an environment. A learning rule (Eq. 4) is proposed to directly minimize orientation estimation error in the network, which implicitly ignores visual features corresponding to less informative or less reliable landmark features, such as non-unique features or features corresponding to local clutter and can form the basis for performant distributed online learning in future robotic investigations.

One of the motivating factors for neural-inspired algorithm development for robotic applications is the potential for power savings and resource efficiency for neuromorphic implementations. Given recent innovations in neuromorphic hardware development with Intel’s Loihi platform^25^, which is able to implement networks with online learning with considerable efficiency, a central challenge in the development of low power neuromorphic robotic applications is to identify performant algorithms utilizing online learning with a compact neural circuit. Conventional algorithms which perform online learning and loop closure with SLAM are power-intense with special-purpose FPGA implementations still requiring approximately 2 or more watts^77,78^. By contrast, conventional approaches without online learning with loop closure, such as VIO, have reduced power consumptions of as little as 2mW on special purpose hardware accelerators^79^. Neuromorphic approaches for robotic navigation show potential for reduced power utilization, such as 9mW in dynamic power consumption demonstrated for a mammalian-inspired network to perform uni-dimensional SLAM with 15,162 compartments^80^. In contrast, our approach utilizes an insect connectivity constrained network with 141 compartments (0.1% of the maximum allowable units on a Loihi chip) and we estimate computation power utilization of 18.6 μW, roughly five orders of magnitude less power than SLAM implementations, and two orders of magnitude less than VIO implementations. While these power estimates are based on published measurements from Loihi SPICE simulations and do not consider system elements such as static power requirements, sensor communication, and low-level visual processing, they nevertheless demonstrate the potential for drastic power savings utilizing inspect-inspired neuromorphic approaches for navigation.

Our modeling results can inform future experiments to better understand the computational mechanisms of heading direction estimation and visually guided navigation in *Drosophila* and the CX more generally. While a hallmark of a typical mammalian head-direction cell is that the preferred direction of the cell is the same regardless of the animal’s location, it is unknown whether a location-invariant heading direction representation is computed in *Drosophila*. In order to perform path integration, a navigation strategy widely observed across insect species^81^ and implicated for *Drosophila*^82^, a location-invariant representation of heading direction relative to a world frame is computationally more robust^81^ . Our modeling results demonstrate that online learning can be used to improve heading direction estimation and path integration error estimates relative to a world frame with a local learning rule, even in the case of position changes through the environment where relative landmark angles change. While the CX-dependent ability to utilize visual features for 2D navigation in an arena whose relative orientations change during movement has been demonstrated experimentally in *Drosophila*^83^, the extent to which the conversion of those visual features to a location-invariant heading direction representation has yet to be tested experimentally. We hypothesize that a local learning which forms a location-invariant heading estimation from visual cues will be a function of input current from multiple neurons, and not only as function of pre- and post-synaptic activity. Additionally, assuming a plasticity rule that drives learning through orientation estimation error rather than co-occurrence of landmark features and heading direction, we predict that the addition of new landmarks after learning should be effectively ignored after an accurate mapping has been developed.

There are several assumptions and simplifications utilized in the presented results. One simplification is that path integration is performed with a known linear velocity. Insect-inspired approaches for path integration in the CX that operate downstream from orientation estimation^74^ are not included in our model. Furthermore, recently released synapse-level neural connectivity data^59,61^ is not incorporated into our model. Another simplification utilized in the network model is the processing of visual features by ring neurons with landmark specific tuning curves. We expect our findings to generalize across more visual feature encoding schemes such as more detailed models of the insect optic lobes, deep networks, or incorporation of processed dynamic vision sensor data which could be investigated in future work. Such encoding schemes could capture visual features that match the expected statistics of landmarks across more realistic environments and provide a richer substrate for learning an orientation mapping.

We present a critical proof of principle for translation of insect-inspired approaches to robotics navigation to enable a future class of low SWaP algorithms to perform online learning and heading representation constrained in detail from biology. Given that all neurons are modeled as dynamic integrate-and-fire neurons, the model is amenable to incorporating event-driven low latency sensors such as dynamic vision sensors to enable updating estimates with visual features detected during high velocity movement. One of the key model features is the ability to utilize previously encountered visual features to update an estimate of orientation, loop closure for orientation estimation, which is not possible in low power navigation approaches utilizing VIO. While this is less than loop closure capabilities of a complete pose in full SLAM systems, it still is a promising functionality due to the ability for the model to be implemented as a parallelized distributed network on neuromorphic hardware. Additionally, compared to deep neural network approaches utilized in visual navigation where networks are trained offline due to computational resource and large data size requirements, the weights in the network model are learned online and are used over a single trajectory to increase estimation accuracy.

In conclusion, we present a critical proof-of-concept for a low SWaP robotics navigation algorithm utilizing orientation estimation in a ring attractor network constrained using circuit details from *Drosophila* with online distributed learning amenable for neuromorphic implementation. By focusing on the objective function minimization necessary for a robotics implementation with a changing position, we establish a formalism for common computational goals underlying both biological and artificial systems and identify testable predictions and areas of focus for future neuroscience experiments.

## Methods

All experiments are performed in a simulated or physical environment utilizing the same connectivity-constrained network model for performing orientation estimation.

### Network model input

**Figure 7:**
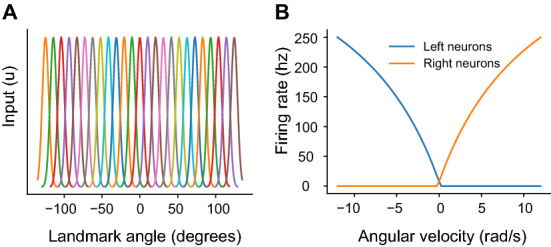
Model input encoding. (**A**) Receptive fields for each set of ring neurons over the field of view. (**B**) Angular velocity encoding in P-EN neurons.

A total of 81 ring neurons are simulated which are selectively tuned to the position of landmarks in the visual field in order to abstract the initial visual processing in the optic lobes. Specifically, sets of 27 ring neurons are selective to each of three landmarks with a Gaussian tuning curve with a standard deviation of 6.44° and a maximum response offset by 10° across a 270° field of view (Fig. 7A). The standard deviation of the Gaussian is selected such that adjacent neurons had overlapping turning curves starting at half maximum values. The current provided to each simulated E-PG neuron in each time step is determined by the visual ring neuron activation multiplied by ${{\varvec{W}}}^{{\varvec{r}}\to {\varvec{c}}}$**.**

The 16 P-EN neurons are split into two hemispheres (right and left), such that the 8 right P-EN neurons encode positive angular velocities, and the 8 left neurons negative velocities. The current provided to each neuron as input is calculated such that with no other input, each P-EN neuron has a steady state maximum firing rate of 250 Hz at 10 radians per second (Fig. 7B). In simulations, zero mean Gaussian noise is added to angular velocity with a standard deviation of 0.1 radians per second.

### Network model and configurations

Neural activation of each ring, E-PG, P-EG, P-EN, and Pintr neuron is modeled as leaky integrate and fire neurons utilizing the nengo software package^84^ with a timestep of 1 ms. The external inputs driving the network activity are input currents from visual encoding to E-PG neurons and angular velocity encoding to P-EN neurons as described above. The connectivity pattern between E-PG, P-EG, P-EN, and Pintr neurons are based on previously reported biologically-constrained connectivity patterns^68^. The network weights between neural subpopulations are 20 for all excitatory connections (E-PG → P-EN, E-PG → P-EG, E-PG → Pintr, P-EN → E-PG, P-EG → E-PG), -15 for all inhibitory connections to excitatory neurons (Pintr → P-EG, Pintr → P-EN), and -20 for all Pintr → Pintr connections. Stochasticity is introduced to the network with mean zero Gaussian noise with a standard deviation of 0.1 added to P-EG and Pintr neurons.

Three model configurations are used whose only difference is the weight of the ring neuron to E-PG connections. For the angular velocity only case, there is no visual input (the effective ${{\varvec{W}}}^{{\varvec{r}}\to {\varvec{c}}}$ is **0**). For the offline optimal comparison, a supervised set of scaled ${{\varvec{W}}}^{{\varvec{r}}\to {\varvec{c}}}$ weights is solved for using linear lasso regularized positive regression to minimize the objective function for each compass neuron ${\sum }_{t}{\left({\tilde{c }}_{n}(t)-{\sum }_{m}(-{r}_{m}(t){\tilde{w }}_{n,m})+{\alpha }_{n}\right)}^{2}+ \lambda {\sum }_{m}{\tilde{w }}_{n,m}$, where ${\tilde{c }}_{n}(t)$ is a target set of compass neuron activation at each simulation timestep generated with the preferred angle of each compass neuron. In order to enforce negative weights for ${w}_{n,m}$, the weights used in simulation are a scaled version of the ${w}_{n,m}=-\beta {\tilde{w }}_{n,m}$, and $\beta$=0.0025, to optimize estimation accuracy. For the online learning rule comparison, the weights between ring neurons and E-PG neurons are updated according to Eq. 1 with parameter values of 1.7e−6, 1.7e−4, and 0.29 for a, b, and $\eta$, respectively.

### Learning rule objective functions

In order to perform gradient descent on the objective function $\phi$ as defined in (Eq. 2) over time by modifying the synaptic weights, $w_{nm}$, it follows from the chain rule that

5 $$\frac{{\partial w_{nm} }}{\partial t} = - \frac{\partial \phi }{{\partial w_{nm} }} = {\text{ a}}c_{n} \frac{{\partial I_{r \to n} }}{{\partial w_{nm} }} - r_{m} \left( {b - w_{nm} } \right) = { }r_{m} \left( {ac_{n} + b - w_{nm} } \right)$$

if we ignore the implicit dependence of $c_{n}$ on $w_{nm}$ assuming that the bump here is determined mostly by the recurrent weights. The discrete form of the gradient descent is

6 $$\Delta w_{nm} = { }\eta \frac{{\partial w_{nm} }}{\partial t} = \eta r_{m} \left( {{\text{a}}c_{n} + {\text{b}} - w_{nm} } \right).$$

Similarly, to minimize the orientation error objective function, ${{\varphi }}$, as defined in (Eq. 3), it follows from the chain rule that

7 $$\frac{{\partial w_{nm} }}{\partial t} = - \frac{\partial \varphi }{{\partial w_{nm} }} = 2{\upbeta }\left( {c_{n} - {\upbeta }I_{r \to n} } \right)\frac{{\partial I_{r \to n} }}{{\partial w_{nm} }} = 2{\upbeta }\left( {c_{n} - {\upbeta }I_{r \to n} } \right)r_{m}$$

with a discrete form of

8 $$\Delta w_{nm} = { }\tilde{\eta }\frac{{\partial w_{nm} }}{\partial t} = { }\eta r_{m} \left( {c_{n} - {\upbeta }I_{r \to n} } \right)$$

w﻿ith a scalar learning rate parameter, $\eta$ = $2{\beta }\tilde{\eta}$.

### Hardware translation

The robotic platform utilized is a modified Robotis Turtlebot3 “Burger,” equipped with a Nvidia Jetson TX2, and a Ricoh Theta S 360° camera. The Turtlebot has an OpenCR embedded motor controller and sensor suite, as well as two Dynamixel XL430-W250 servos for wheel control.

The relative angle of colored landmarks in the arena are detected from 360° camera images utilizing color masks and blob detection prior to activation of visual ring neurons as described above with Gaussian receptive fields with respect to the relative angle of each landmark. All sensor data was recorded in ROS and used as input to the network model. We accelerated the input stream by a factor of ten to facilitate an evaluation of time courses corresponding to those used in simulated environments.

The robot was run in an arena with an Optitrack system. IR-reflective markers were attached to the robot such that the position and orientation of the robot was tracked by the system. Red, green, blue, and yellow landmarks were made out of posterboard and placed in the environment with their own IR markers. Data from the Optitrack system was used for comparison.

### Power estimation

The estimated power consumption for algorithmic computation utilizes published energy per operation values^25^ and is calculated seperately for the neuron update, (${e}_{neuron}={n}_{neuron}*81\text{ pJ}$), spike communication (${e}_{comm}=\frac{1}{T}\sum_{t=1}^{T}\sum_{j=1}^{{n}_{neuron}}\sum_{i=1}^{{n}_{neuron}}{y}_{i}(t){w}_{i,j}^{+}*1.7 {\text{pJ}}$), and plasticity (${e}_{plasicity}={n}_{synRC}*120 {\text{pJ}}/\mathrm{\Delta p}$), where ${n}_{neuron}$ is the total number of neurons in the network, $T$ is the total number of timesteps, ${y}_{i}(t)$ is the binary spike output for each neuron in each time step $t$, ${w}_{i,j}^{+}$ is a binary variable equal to 1 if there is a non-zero weight from neuron $i$ to neuron $j$, ${n}_{synRC}$ is the total number of synapses between ring and E-PG neurons, and the and ${\Delta \mathrm{p}}$ is the number of time steps assumed to be 63 between synaptic update. The final power estimates assume 1000 time steps per second.

## Data Availability

All data needed to support the conclusions in the paper are available via sources described in the paper or upon reasonable request to the authors.
