# Author Correction: Online learning for orientation estimation during translation in an insect ring attractor network

**DOI:** 10.1038/s41598-022-08814-9

**Published:** 2022-03-18

**Authors:** Brian S. Robinson, Raphael Norman-Tenazas, Martha Cervantes, Danilo Symonette, Erik C. Johnson, Justin Joyce, Patricia K. Rivlin, Grace M. Hwang, Kechen Zhang, William Gray-Roncal

**Affiliations:** 1 The Johns Hopkins University Applied Physics Laboratory, Laurel, MD 20723 USA; 2 Janelia Research Campus, Howard Hughes Medical Institute, Ashburn, VA 20147 USA; 3 Kavli Neuroscience Discovery Institute, Johns Hopkins University, Baltimore, MD 21205 USA; 4 Department of Biomedical Engineering, Johns Hopkins University, Baltimore, MD 21205 USA; 5 Department of Neuroscience, Johns Hopkins University, Baltimore, MD 21205 USA; 6 Department of Computer Science, Johns Hopkins University, Baltimore, MD 21218 USA

Correction to: *Scientific Reports* 10.1038/s41598-022-05798-4, published online 25 February 2022

The original version of this Article contained an error in the spelling of the author Grace M. Hwang, which was incorrectly given as Grace Hwang.

The original Article has been corrected.

